# Postembryonic development and male paedomorphosis in *Osedax* (Siboglinidae, Annelida)

**DOI:** 10.3389/fnins.2024.1369274

**Published:** 2024-03-18

**Authors:** Katrine Worsaae, Alice Rouan, Elaine Seaver, Norio Miyamoto, Ekin Tilic

**Affiliations:** ^1^Marine Biological Section, Department of Biology, University of Copenhagen, Copenhagen, Denmark; ^2^The Whitney Laboratory for Marine Bioscience, University of Florida, Gainesville, FL, United States; ^3^X-STAR, Japan Agency for Marine-Earth Science and Technology, Yokosuka, Japan; ^4^Department of Marine Zoology, Senckenberg Research Institute and Natural History Museum, Frankfurt, Germany

**Keywords:** developmental staging, neurogenesis, myogenesis, trochophore, metamorphosis, male dwarfism

## Abstract

Most species of the bone-devouring marine annelid, *Osedax,* display distinct sexual dimorphism with macroscopic sedentary females rooted in bones and free-living microscopic dwarf males. The paedomorphic male resembles the non-feeding metatrochophore larva in size, presence of eight pairs of chaetae, and a head ciliation potentially representing a residual prototroch. The male development may thus uniquely reiterate and validate the theoretical heterochrony process “progenesis”, which suggests that an accelerated sexual maturation and early arrest of somatic growth can lead to a miniaturized and paedomorphic adult. In this study, we describe the postembryonic larval and juvenile organogenesis of *Osedax japonicus* to test for a potential synchronous arrest of somatic growth during male development. Five postembryonic stages could be distinguished, resembling day one to five in the larval development at 10°C: (0D) first cleavage of fertilized eggs (embryos undergo unequal spiral cleavage), (1D) pre-trochophore, with apical organ, (2D) early trochophore, + prototroch, brain, circumesophageal connectives and subesophageal commissure, (3D) trochophore, + telotroch, four ventral nerves, (4D) early metatrochophore, + protonephridia, dorsal and terminal sensory organs, (5D) metatrochophore, + two ventral paratrochs, mid-ventral nerve, posterior trunk commissure, two dorsal nerves; competent for metamorphosis. The larval development largely mirrors that of other lecithotrophic annelid larvae but does not show continuous chaetogenesis or full gut development. Additionally, *O. japonicus* larvae exhibit an unpaired, mid-dorsal, sensory organ. Female individuals shed their larval traits during metamorphosis and continue organogenesis (including circulatory system) and extensive growth for 2–3 weeks before developing oocytes. In contrast, males develop sperm within a day of metamorphosis and display a synchronous metamorphic arrest in neural and muscular development, retaining a large portion of larval features post metamorphosis. Our findings hereby substantiate male miniaturization in *Osedax* to be the outcome of an early and synchronous offset of somatic development, fitting the theoretical process “progenesis”. This may be the first compelling morpho-developmental exemplification of a progenetic origin of a microscopic body plan. The presented morphological staging system will further serve as a framework for future examination of molecular patterns and pathways determining *Osedax* development.

## 1 Introduction

### 1.1 Progenesis as a pathway to miniature body size and dwarf males

There is an increasing awareness that evolution does not always lead to larger, more complex organisms; it can also result in drastic decreases in body size and complexity. An obvious advantage of miniaturization is a reduced nutritional and spatial demand, which can potentially lessen competition between sexes in species with dwarf males (Vollrath, 1998) and provide access to new niches, such as the interstices between sand grains inhabited by microscopic interstitial taxa (Westheide, 1987). These “underdeveloped” miniature body forms, which may exhibit juvenile or larval (paedomorphic) characteristics, are often hypothesized to be the outcome of heterochrony. This refers to a change in the timing or rate of development relative to the ancestor, such as an early offset of somatic growth (progenesis; Garstang, 1922; Gould, 1977; Smith, 2001; Worsaae and Kristensen, 2005; McNamara, 2012; Martynov et al., 2020). Progenesis has been a particularly popular theory for explaining the origin of numerous meiofauna lineages phylogenetically positioned within otherwise macrofaunal groups (e.g., Westheide, 1987; Laumer et al., 2015; Struck et al., 2015; Martín-Durán et al., 2020; Worsaae et al., 2021, 2023). It also has been proposed to account for the origin of paedomorphic dwarf males in species with significantly larger females (e.g., Schuchert and Rieger, 1990; Vollrath, 1998; Rouse et al., 2008; Worsaae and Rouse, 2010). Progenesis is suggested to be linked to, or even driven by, early or accelerated sexual maturation, leading to the simultaneous arrest of somatic growth and organogenesis (Gould, 1977; Alberch et al., 1979; Westheide, 1987; Raff and Wray, 1989). However, due to the lack of fossil evidence of ancestors and the significant evolutionary distance from even the closest macroscopic relative, it is rarely possible to provide morphological evidence for this theoretical process. Ideally, such evidence would demonstrate a detailed anatomical resemblance between the miniaturized organism and an earlier developmental stage of its ancestor or a close relative (Westheide, 1987; Worsaae and Kristensen, 2005; Struck, 2006; Kerbl et al., 2016; Worsaae et al., 2023). Developmental studies of species with paedomorphic dwarf males may provide a unique opportunity to document progenesis, as detailed morphological and molecular comparisons between males and females are more feasible than between evolutionarily distant lineages. Such thorough morphological documentation is crucial, not only for substantiating this theory, but also as a platform for identifying the specific developmental and genomic mechanisms causing these evolutionary changes.

Sexual dimorphism in the form of dwarf males can reduce competition between sexes in habitats with limited food or space, or optimize fertilization in species with small mating group size (Vollrath, 1998; Urano et al., 2009). Dimorphism is thus often observed in species with space-limited, sedentary females, such as in various spiders (Vollrath, 1998), Cirripedia (e.g., Lin et al., 2015), Cycliophora (Obst and Funch, 2003), and the annelids *Osedax* (Rouse et al., 2004, 2008; Worsaae and Rouse, 2010) and *Bonnelia* (Schuchert and Rieger, 1990). However, it also occurs in free-living species with “over dispersed” population structures, including deep-sea anglerfish (Bertelsen, 1951), the meiofaunal annelid *Dimorphilus gyrociliatus* (Schmidt, 1857) (Schmidt, 1857; Windoffer and Westheide, 1988a,b), monogonont Rotifera (Wesenberg-Lund, 1923, 1930; Ricci and Melone, 1998; Gąsiorowski et al., 2019), the free-living stages of the parasitic Orthonectida (Slyusarev et al., 2018, 2023). In some cases, like for the orb-weaving spider *Nephila*, phylogenetic tracing has shown that their pronounced dimorphism is likely due to female gigantism rather than male dwarfism (Coddington et al., 1997). *Osedax* is the only genus in Siboglinidae that displays sexual dimorphism, which suggests that male dwarfism is an apomorphic trait of *Osedax*. *Osedax* females have a much more complex morphology, with four palps, a trunk that can extend several centimeters in length, and a root system penetrating their preferred bony substrate and housing symbiotic bacteria (Rouse et al., 2004; Vrijenhoek et al., 2009; Rouse et al., 2018). In contrast, males are of microscopic size and non-feeding (Worsaae and Rouse, 2010). Moreover, deeply nested within the *Osedax* clade, the exceptional *O. priapus* Rouse, Wilson, Worsaae & Vrijenhoek, 2015 (Rouse et al., 2015) has evolved large males with symbiotic bacteria, roots, a long trunk, and two palps and hereby exemplify secondary gain of macroscopic male size and sexual monomorphism (Rouse et al., 2015). In *Osedax*, sexual dimorphism is undeniably a result of male dwarfism. Dwarf males have been described as paedomorphic due to their superficial resemblance to the larvae in size, presence of anterior ciliation, and a similar number of chaetae (e.g., Rouse et al., 2009; Worsaae and Rouse, 2010). However, to ascertain whether and how male dwarfism in *Osedax* is a consequence of progenesis, detailed studies of larval development and ontogenesis are necessary.

### 1.2 Larval development in annelids

Both planktotrophic (feeding) and lecitotrophic (non-feeding) larvae, as well as direct development exist in Annelida. The evolution of the trochophore larva is still debated (Rouse, 1999; Nielsen, 2018). However, all annelid trochophores show a characteristic equatorial ciliary band (prototroch) used for swimming, typically supported by a posterior band (telotroch). Both bands consist of compound cilia that beat in metachronal waves. A metatroch ring is found in many, though not all, planktotrophic trochophores (Bleidorn et al., 2015). This ring, along with the prototroch, and in some cases a midventral neurotroch, assists in particle capture (Nielsen, 2004). The initial larval stages are termed “protrochophore,” followed by the early, mid, and late “trochophore” stages. For older larvae with a segmented body, the terms early, mid, and late “metatrochophore” are used. The term “nectochaeta” refers to pelagic larvae that move with the aid of parapodia and chaetae (e.g., Fischer et al., 2010). Moreover, various aberrant late larval stages have been given distinct terminology, such as the “pericalymma” larva that already develops part of the adult body, found in, e.g., *Owenia* and *Polygordius* (Nielsen, 2004, 2018; Helm et al., 2016; Carrillo-Baltodano et al., 2021). The most extensively studied annelid larvae are those of the model organisms *Capitella teleta* Blake, Grassle and Eckelbarger, 2009 (Sedentaria, Annelida) (Blake et al., 2009), which has six postembryonic lecithotrophic larval stages (stages 4–9 in Seaver et al., 2005), and *Platynereis dumerilii* (Audouin and Milne Edwards, 1833) (Errantia, Annelida) (Audouin and Milne Edwards, 1833), with 10 postembryonic planktotrophic larval stages (Fischer et al., 2010). However, annelids display a vast diversity of larval morphologies and developmental processes. Detailed studies of organogenesis, particularly neurogenesis, have been conducted for only a limited number of species, and no studies have focused on members of the family Siboglinidae (e.g., Bullock and Horridge, 1965; Lacalli, 1984; Dorresteijn et al., 1993; Hay-Schmidt, 1995; Voronezhskaya et al., 2003; Orrhage and Müller, 2005; McDougall et al., 2006; Brinkmann and Wanninger, 2008; Pernet and McHugh, 2010; Helm et al., 2013, 2016; Meyer et al., 2015; Pernet et al., 2015; Kerbl et al., 2016; Starunov et al., 2017; Carrillo-Baltodano et al., 2019, 2021).

### 1.3 *Osedax* development and paedomorphic males

*Osedax japonicus* Fujikura, Fujiwara & Kawato, 2006 (Fujikura et al., 2006) is one of the few species of Siboglinidae for which a long-term laboratory culture has been successfully established, enabling the study of its the entire life cycle in a laboratory setting (Miyamoto et al., 2013, 2017). However, detailed descriptions of its larval development and ontogenesis have not yet been provided. Thus, there remains a lack of comprehensive descriptions of neurogenesis within the family Siboglinidae, with only a few studies addressing siboglinid larval morphologies (Nørrevang, 1970; Bakke, 1977; Gardiner and Jones, 1994; Callsen-Cencic and Flügel, 1995; Bright et al., 2013; Rimskaya-Korsakova et al., 2021; Temereva and Rimskaya-Korsakova, 2023). The post-embryonic development of *Osedax* has been briefly described, primarily based on light microscopy observations for *O. packardorum* Rouse, Goffredi, Johnson & Vrijenhoek, 2018 (Rouse et al., 2018) (as *O*. “orange collar” in Rouse et al., 2009) and *O. japonicus* (Miyamoto et al., 2013). Additionally, coherent observations of individual life stages have been reported for other *Osedax* species (e.g., Rouse et al., 2009). These studies have documented a lecithotrophic development with unequal spiral cleavage in the embryonic stages, and a relatively simple-looking, non-feeding, swimming trochophore larva. This larva possesses an apical tuft, a prototroch, a telotroch, as well as a metatrochophore stage with 16 posterior chaetae. Both Rouse et al. (2009) and Miyamoto et al. (2013) indicated a relatively short life span for the larvae and the absence of a nectochaete stage. Although the larvae superficially resemble the dwarf males in size and presence of chaetae, their ciliary patterns and body forms are somewhat different, and the internal anatomy has not been compared or described throughout development and metamorphosis into males.

In this work, we describe the postembryonic development and neurogenesis of *O. japonicus*, characterizing the larval stages and the metamorphosis into both males and females. We employ light microscopy, immunohistochemistry and CLSM. Through the comparison of larval and adult anatomy, we aim to identify the potential progenetic origin of *Osedax* dwarf males.

## 2 Materials and methods

### 2.1 Animal collection

The animal study was carried out on a laboratory culture of a non-endangered marine annelid species, *Osedax japonicus* (see Miyamoto et al., 2013, 2017). *Osedax japonicus* females were kept at 10°C on vertebrate bones (for more details see Miyamoto et al., 2013, 2017). Mucus surrounding the palps was transferred to filtered artificial sea water (ASW) and fertilized oocytes were isolated from mucus and kept at 10°C in filtered ASW. Time was recorded from the first cleavage (2 cell stage) as hours post-cleavage (hpc). Batches of embryos and larvae were kept in filtered ASW and fixed at scheduled times throughout development. In order to induce metamorphosis, +5-day-old competent larvae were exposed to *Pagrus major* (sea bream) scales (previously stored in ethanol and washed) and incubated with an *Oceanospirillales* sp. culture. Early stage males and females were fixed after at least 9 h post induction (hpi), and were morphologically distinguishable by this time. Females were allowed to develop on the fish scales and were sampled after 1.5–4 days post-induction, when they had two distinguishable palps, and after 1–3 weeks, when they had 4 fully grown palps. The late stage males were fixed 4–9 days post-induction of metamorphosis.

### 2.2 Fixation

Animals were anesthetized in 7% magnesium chloride (MgCl_2_) in 1:1 ratio with seawater. Females (two palp and four palp stages) were either dissected from the scale after anesthesia or fixed on scales. Samples were fixed in 4% paraformaldehyde (Electron Microscopy Sciences, 15,710) in phosphate-buffered saline solution (PBS) with 7% sucrose for an hour at room temperature (RT) or overnight at 4°C (Worsaae et al., 2016). Samples were rinsed six times in PBS (with 7% sucrose) and stored in PBS (with 7% sucrose, 0.05% NaN_3_) for F-actin staining or in 100% methanol (MeOH) following washes gradually increasing MeOH concentrations.

### 2.3 Light microscopy and behavioral observations

To observe swimming behaviors, 2-day old larvae to 5-day old competent larvae were observed using a dissecting microscope. The light microscopy images and videos were acquired using an inverted compound microscope (OLYMPUS, IX 71) with a mounted camera (Nikon, D7200) examining animals in small petri dishes with coverslip bottom or mounted on slides in filtered ASW, sometimes adding 7% MgCl_2_ solution for further immobilization.

### 2.4 Immunohistochemistry and CLSM

Ciliary, nervous and muscular systems were examined with immunohistochemical and direct multi-staining protocols as described in Kerbl et al. (2016), using F-actin staining (Alexa Fluor 488-labeled phalloidin, INVITROGEN, A12379), DNA-staining [405-DAPI in vectashield mounting system (Sigma-Aldrich, H-1200)] and immunostaining (monoclonal mouse acetylated α-tubulin (Sigma-Aldrich, T6793), anti FMRFamide [Immunostar, 20,091) and polyclonal rabbit anti-serotonin (5-HT, Sigma-Aldrich S5545)]. Samples stored in MeOH were rehydrated in decreasing concentrations of MeOH (95%, 75%, 50%, 25%). Specimens were pre-incubated for 4-6 h in PTA (PBS with 5% Triton-X, 0.25% BSA, 7% sucrose) prior to 48 h incubation on a rocking table at RT in primary antibodies mixed 1:1 in PTA 0.1% (1,400 final concentration). This was followed by 4 to 6 rinses of 15–30 min in PTA 0.1% before a 2 h incubation in PTA 0.1% + 5% goat serum. Subsequently, samples were incubated on a rocking table in two secondary antibodies mixed 1:1 in PTA 0.1% (1:400 final concentration, sheep anti-rabbit CY3 [Sigma-Aldrich, C2306), goat anti-mouse Cy5 (Jackson ImmunoResearch, 115-175-062)]. After 24 h, phalloidin was added (0.33 μM) to the secondary antibody mix for a total incubation of 48 h. Samples were then washed 4 times for 30 min each in PBS (PBS 1X, 7% sucrose) and gradually raised to 100% mounting media with DAPI before being individually mounted on slides.

The slides were scanned on an OLYMPUS IX 81 inverted confocal microscope with a Fluoview FV-1000. Acquired z-stacks were analyzed with Imaris 7.0 and/or Imaris viewer 10.0 (Bitplane Scientific Software) and maximum intensity z-projections images were created from cropped or full z-stacks. Plates and drawings were assembled on Adobe Illustrator v28.2 (2024) and Inkscape v1.3.1.

## 3 Results

### 3.1 Overview of *Osedax japonicus* development

**Figure 1:**
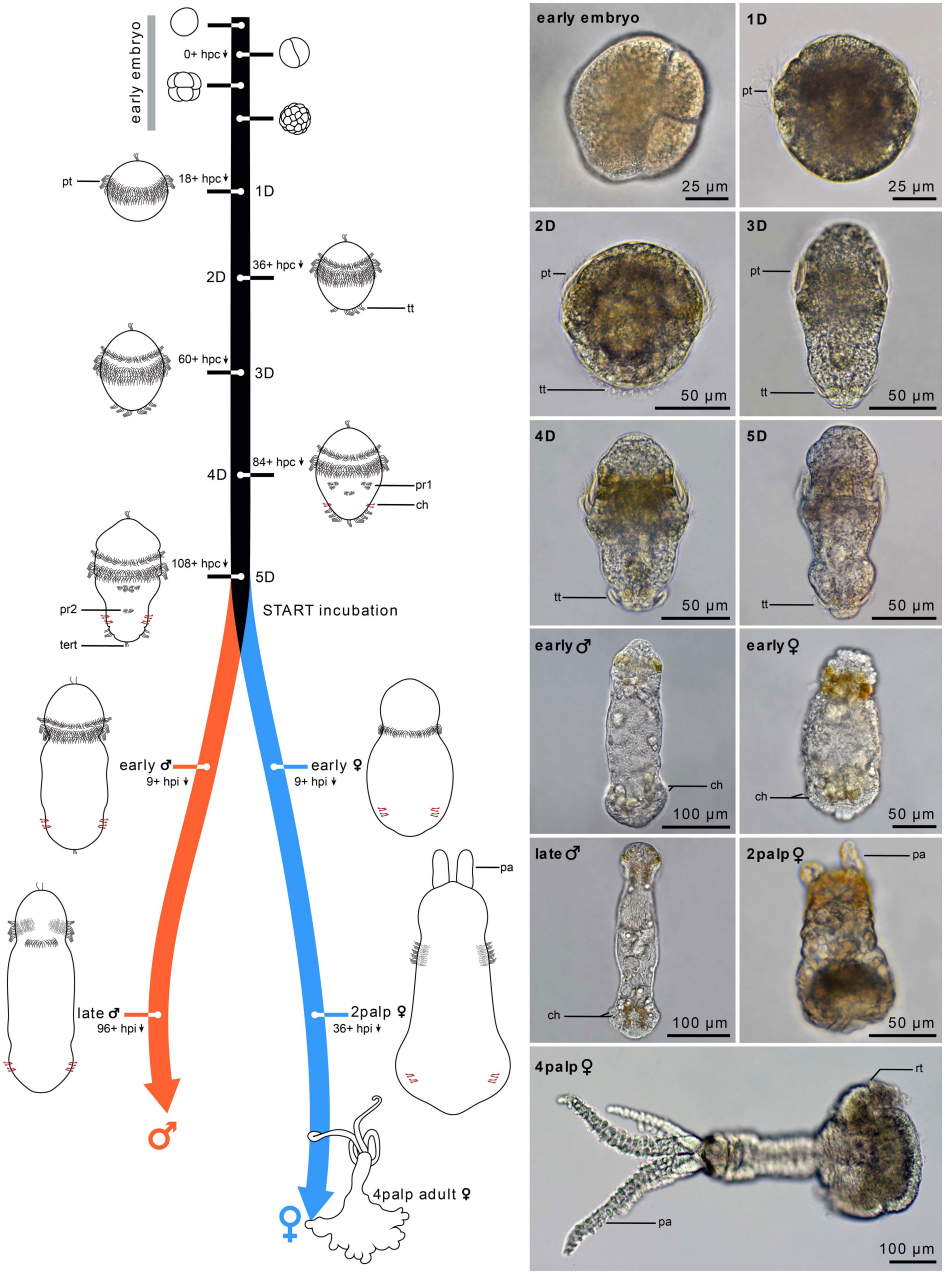
Timeline of *Osedax japonicus* development. Schematic drawings of embryonic development (0–24 h) and postembryonic development from 1-day-old (1D) larva to 5-day-old (5D) larva. The right panel presents light microscopy (LM) images of the different stages, with the development of two-cell-row ciliated prototroch (1D larva), followed by completion of prototroch with third row of cells and formation of telotroch (2D larva), separation of anterior prototroch row (3D larva), onset of medio-ventral paratroch 1 and formation of chaetae (4D larva), and appearance of paratroch 2 (5D larva). Upon incubation with cues, larvae metamorphose into early females or early males (9 h post incubation). The females develop into a 2-palps stage (1.5 days to 4 days post incubation) and finally into a 4-palps adult female (2–3 weeks), while males reach maturity after only 4 to 8 days post incubation. 1D–5D, 1–5 day-old larva; ch, chaetae; hpc, hours post cleavage; hpi, hours post incubation; pa, palp; pt, prototroch; pr, paratroch; rt, root; tert, terminal tuft; tt, telotroch.

We here describe the postembryonic development of *O. japonicus* and introduce an effective staging system largely identifiable with light microscopy (LM; Figure 1), which includes five larval stages preceding metamorphosis as well as 2 male and 3 female post metamorphic stages. Adding to a previous study of Miyamoto et al. (2013), we characterize the detailed development and organogenesis using both live behavioral and detailed morphological observations, employing immunohistochemistry labeling and CLSM—with specific emphasis on the neural development.

**Figure 2:**
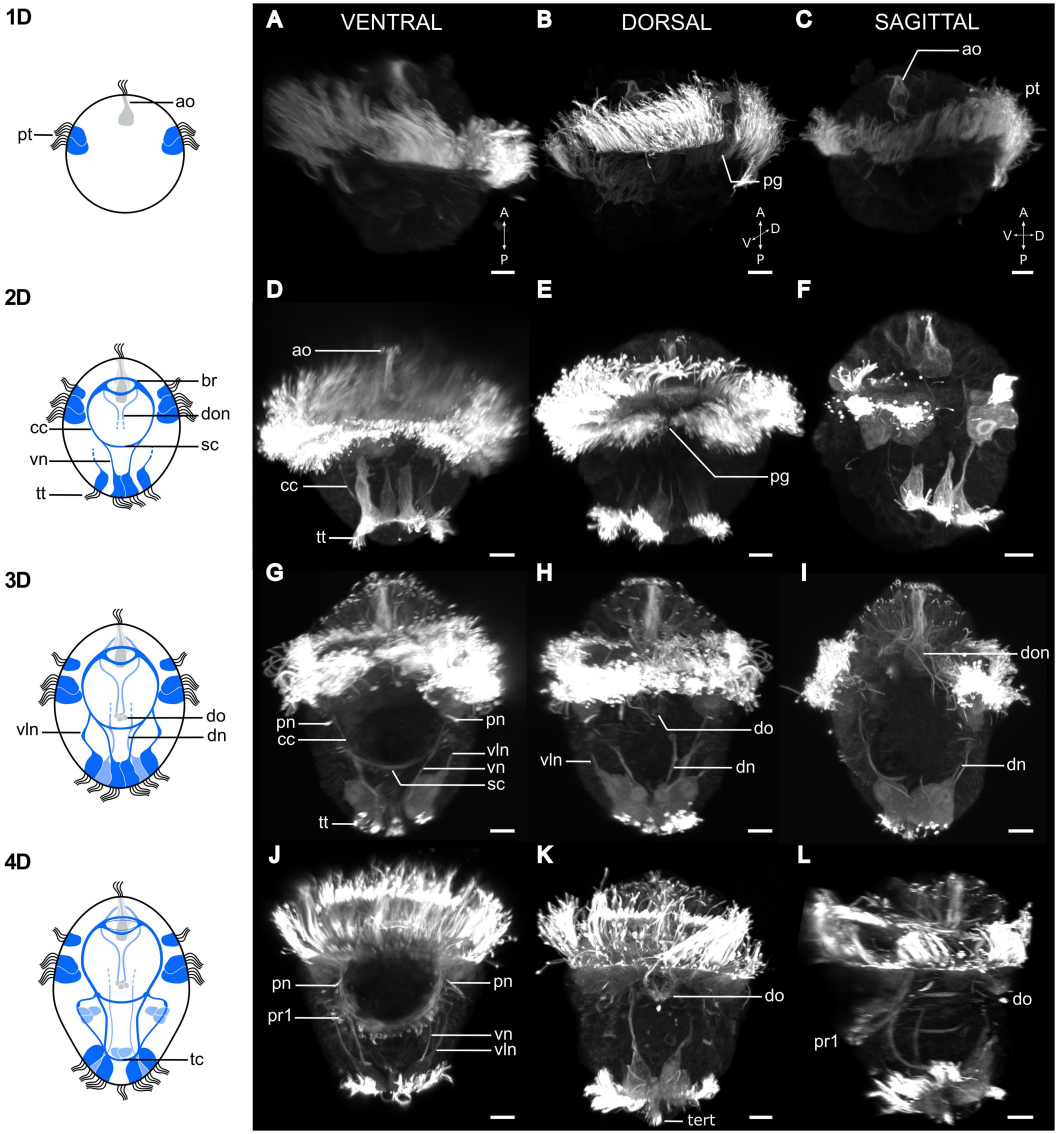
Postembryonic development of *Osedax japonicus* nervous system. Left panel shows schematic drawings of the nervous system from 1 to 4 day old larvae (1D–4D) based on acetylated α-tubulin-LIR, imaged by CLSM and displayed in the right panels **(A,D,G,J)**. Ventral view of maximum intensity z-projection with some virtual sectioning to remove dorsal and/or ventral prototroch signal. **(B,E,H,K)** Dorsal view of maximum intensity z-projection with some virtual sectioning to remove ventral signal. **(C,F,I,L)** Ventro-dorsal sagittal virtual section of maximum intensity z-projection, with both sides of lateral prototroch virtually sectioned. **(A–C)** Image of 1D larva with prototroch ring and flask-shaped cell of apical organ. **(B)** Slightly rotated from dorsal view to better show dorsal prototroch gap. **(D–F)** 2D Larva showing emergence of brain, circumesophageal connectives, ventral nerves, and extension of ventrolateral nerves. Telotroch cells form incomplete ring on ventral side and prototroch gap remains open on dorsal side. **(G–I)** Image of 3D larva with ventral protonephridia, development of dorsal organ, and different dorsal nerves. Prototroch is closed, but telotroch remains open on ventral side. **(J–L)** 4D larva exhibiting line of paratroch 1 cells along subesophageal commissure, joined by ventral and ventrolateral nerves. Telotroch ring closed, terminal tuft visible. Color codes: *dark blue* ventral nervous system structure, *light blue* dorsal nervous system structure. 1D, 1 day-old larva; a, anterior; ao, apical organ; br, brain; cc, circumesophageal connectives; D, dorsal; dn, dorsal nerves; do, dorsal organ; don, dorsal organ nerves; P, posterior; pg, prototroch gap; pn, protonephridia; pt, prototroch; pr1, paratroch 1; sc, subesophageal commissure; tc, terminal commissure; tert, terminal tuft; tt, telotroch; V, ventral; vln, ventro-lateral nerves; vn, ventral nerves. Scale bars represent 10 μm.

**Figure 3:**
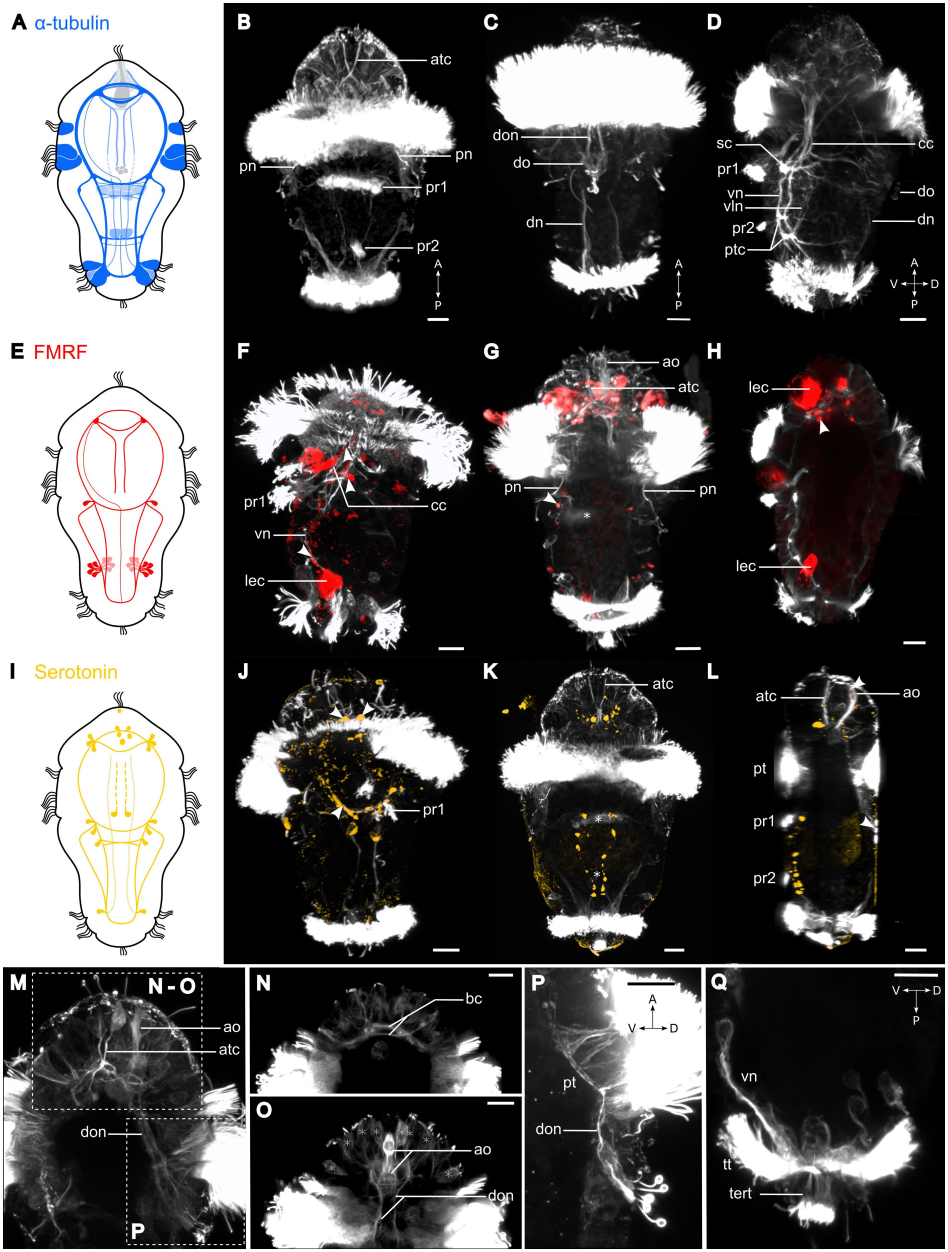
Nervous system of *Osedax japonicus* 5-day-old competent larva. (**A,E,I**) Schematic drawing of 5-day-old larva (5D) with acetylated α-tubulin-LIR (ventral in *dark blue* and dorsal in *light blue*), FMRFamide-LIR (*red*), and Serotonin-LIR (*yellow*). (**B–D,F–H,J–Q**) Maximum intensity z-projection of CLSM scans with acetylated α-tubulin (*white*), **(F–H)** FMRFamide (*red*), **(J–L)** serotonin (*yellow*). Projections were virtually sectioned to remove either dorsal, ventral, or prototroch signals for better display of intended structures. **(B)** Ventral view of acetylated α-tubulin-LIR. Two fully formed ventral paratrochs, two apical α-tubulin-LIR cells with stiff external cilia. **(C)** Dorsal view of two dorsal organ nerves and dorsal organ. **(D)** Ventro-dorsal view with virtual sectioning of lateral prototroch, displaying circumesophageal connectives (*cc*) connecting to subesophageal commissure (*sc*), and ventral and ventrolateral nerves connected by posterior trunk commissure. **(F)** Ventro-dorsal view of acetylated α-tubulin-LIR and FMRFamide-LIR. Arrows pointing at FMRFamide-LIR along *cc* and at sc junction. **(G)** Ventral view with few ventral sections virtually removed, showing paratroch 1 (*) and FMRFamide-LIR in lateralsomata at *cc* and *sc* connection (arrow). **(H)** Ventro-dorsal view of FMRFamide-LIR in brain. **(J)** Ventral view of acetylated α-tubulin-LIR and serotonin-LIR in ventral brain (anterior arrows), *cc* and *sc* (posterior arrows). **(K)** Ventral view of serotonin-LIR in somata along *vn* and in brain around apical organ. *Paratrochs 1, 2* are localized with *. **(L)** Ventro-dorsal view showing serotonin-LIR in the apical and dorsal organs (arrows). **(M)** Detailed ventro-dorsal view of acetylated α-tubulin-LIR in anterior and dorsal part. **(N)** Detailed apical region in ventral view showing brain commissures. **(O)** Detailed apical region in dorsal view displaying apical organ and dorsal organ nerves extending to brain. Potential sensory cells of apical plate (*). **(P)** Detailed lateral view of dorsal organ and dorsal organ nerves connecting to brain and prototroch cells. **(Q)** Detailed posterior region in lateral view with virtual sectioning showing terminal tuft and telotroch. a, anterior; ao, apical organ; atc, apical α-tubulin-LIR cell; bc, brain commissure; cc, circumesophageal connectives; D, dorsal; dn, dorsal nerves; do, dorsal organ; don, dorsal organ nerves; lec, large-bodied epidermal cells; P, posterior; pn, protonephridia; pt, prototroch; ptc, posterior trunk commissures; pr, paratroch; sc, subesophageal commissure; tert, terminal tuft; tt, telotroch; V, ventral; vln, ventro-lateral nerves; vn, ventral nerves. Scale bars represent 10 μm.

Fertilized eggs of *O. japonicus* are continuously released by the female and most of these are trapped in the mucus holster surrounding her trunk. Within a few days at 10°C, the embryos develop into a non-feeding (lecithotrophic) trochophore larva (Figure 2). The 5-day old metatrochophore larva is already competent for metamorphosis but can remain as a larva for at least 3 weeks at 10°C in absence of environmental metamorphic cues. The metatrochophore shows characteristic annelid larval traits such as paired protonephridia, chaetae, a ciliated “gut” structure (in potential female larvae), an apical organ, a two-commissural brain and a pentaneural nerve cord (Figure 3). Noteworthy neurodevelopmental events include the differentiation of the first neurons in the brain with subsequent growth posteriorly, and the later formation of the five ventral and two dorsal longitudinal neurite bundles (nerves) with supra-esophageal (brain), subesophageal, posterior trunk, and terminal commissures.

**Figure 4:**
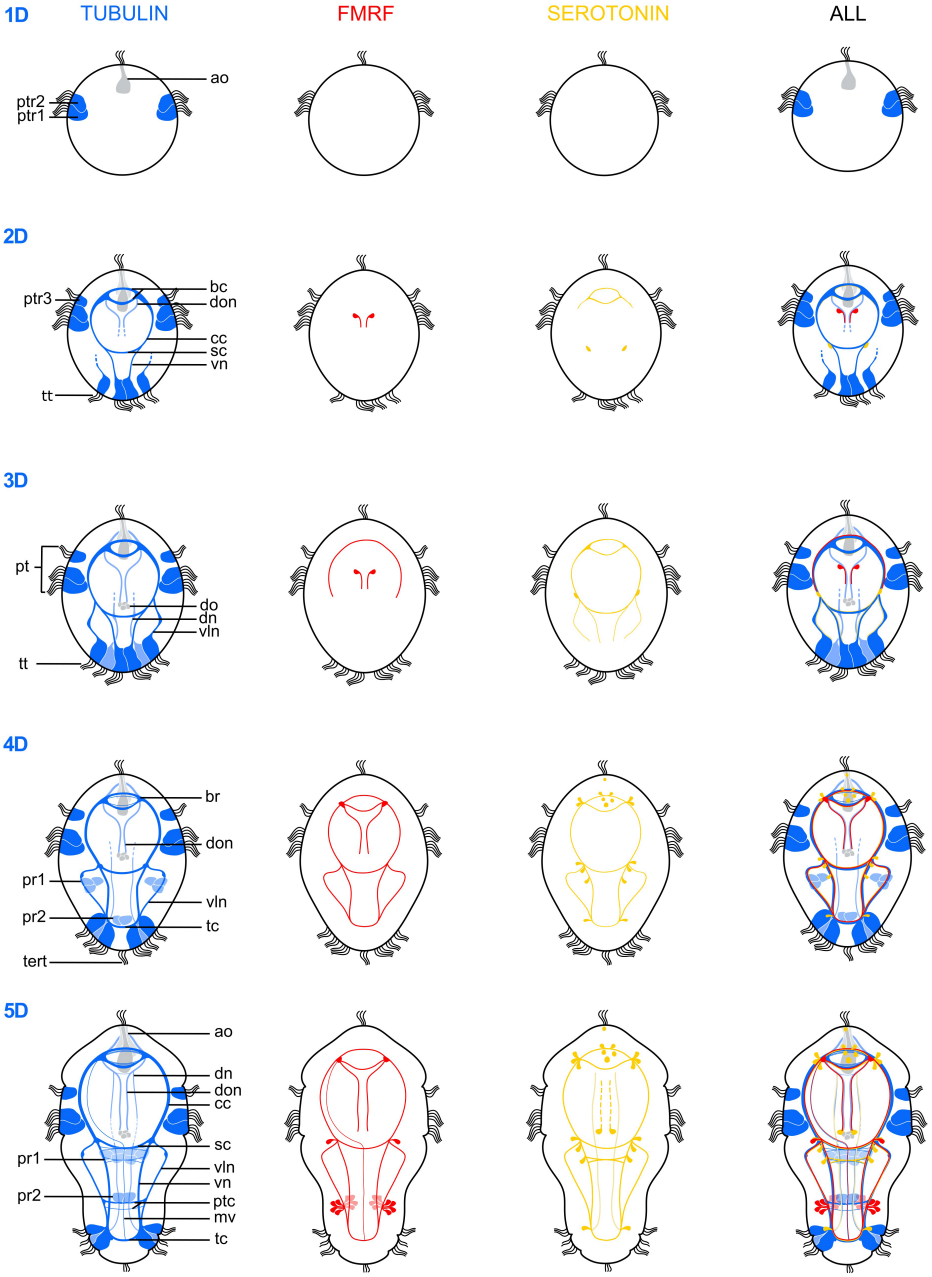
Schematic drawings illustrating the postembryonic nervous system development of *Osedax japonicus* from a 1-day-old larva (1D) to a 5-day-old (5D) competent larva. The development is depicted based on acetylated α-tubulin-LIR (left, *dark blue* ventral, *light blue* dorsal), FMRFamide-LIR (middle-left, *red*), Serotonin-LIR (middle-right, *yellow*) and a combined view (right). ao, apical organ; bc, brain commissure; br, brain; cc, circumesophageal connectives; dn, dorsal nerves; do, dorsal organ; don, dorsal organ nerves; mv, unpaired medio-ventral nerve; pt, prototroch; ptc, posterior trunk commissures; ptr, prototroch row; pr, paratroch; sc, subesophageal commissure; tc, terminal commissure; tert, terminla tuft; tt, telotroch; vln, ventro-lateral nerves; vn, ventral nerves.

**Figure 5:**
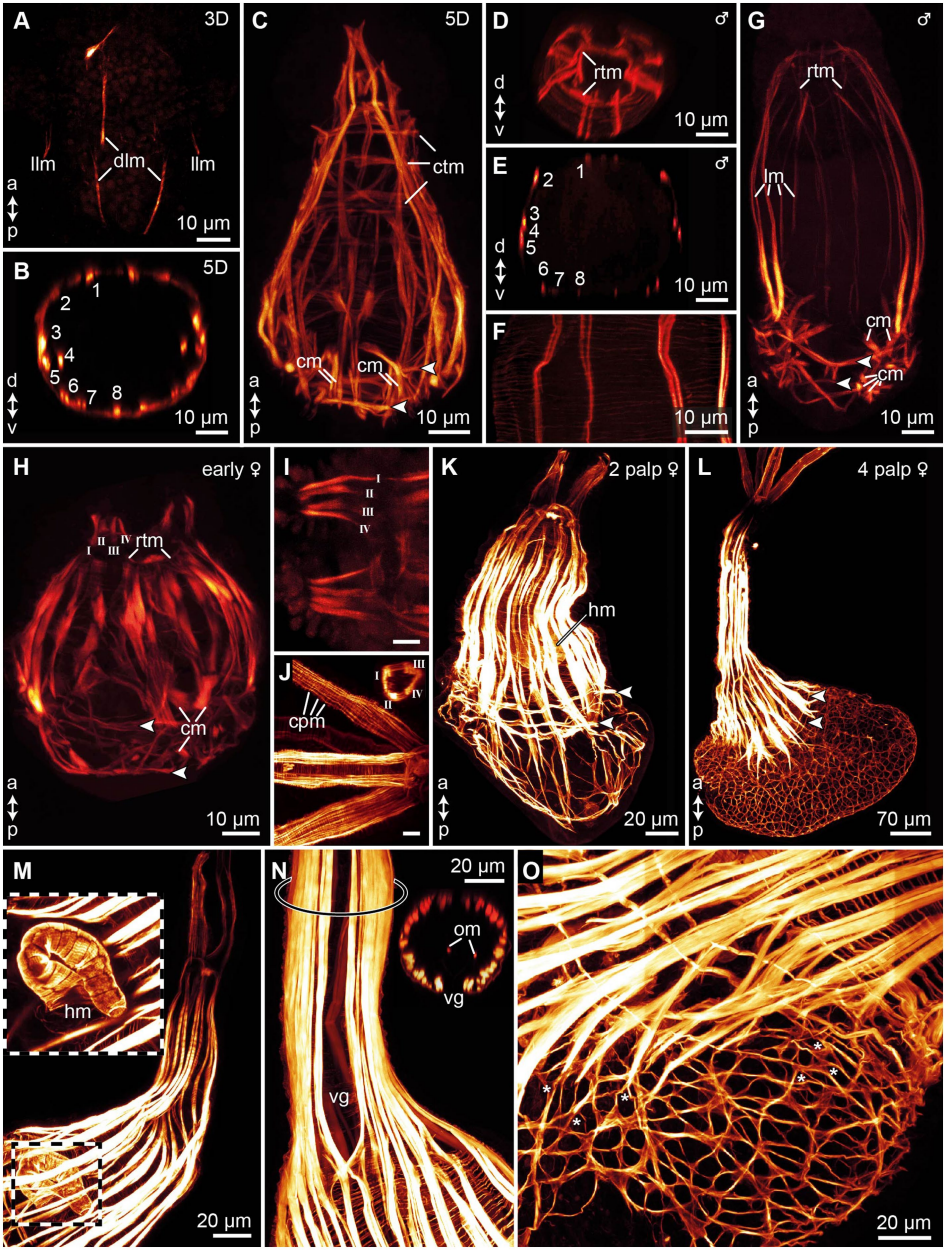
Myogenesis in *Osedax japonicus*, maximum intensity z-projections images of CLSM scans of phalloidin stained musculature. **(A)** 3-day-old (3D) larva showing initial emergence of longitudinal muscle fibers. **(B)** Virtual cross-section of 5D larva, illustrating arrangement and number of longitudinal muscles. **(C)** Confocal Z-projection of 5D larva, highlighting longitudinal muscles tapering apically to connect with the forming apical ring of transverse muscles (*rtm*), and the thin circular transverse muscles, including two prominent transverse muscle rings connected to chaetae retractors, indicated by arrowheads in all panels. **(D)** Virtual cross-section through apical region of dwarf male, depicting *rtm*. **(E)** Virtual cross-section of male, showing arrangement and number of longitudinal muscles. **(F)** Detailed view of body wall in male, revealing thin meshwork formed by circular transverse musculature. **(G)** Z-projection of entire male specimen. **(H)** Confocal Z-projection of early-stage female with two budding palps; longitudinal palp muscles labeled using Roman numerals. **(I)** Detailed view of palp musculature in early-stage female with two palps; anterior end to the left. **(J)** Detailed view of palp musculature in female with four palps, anterior end to the left. Insert showing cross-section of palp. **(K)** Confocal Z-projection of female with two palps. **(L)** Confocal Z-projection of female with four palps. **(M)** Image of trunk and heart musculature in female with four palps, with outer muscle layers digitally removed to expose internal heart structure. **(N)** Z-projection of trunk in female with four palps, displaying ventral gap in longitudinal trunk muscles and two internal longitudinal oviduct muscles, with location of inserted cross-section marked by ring on trunk. **(O)** Trunk-root transition in female with four palps, showing root musculature and branching points of some longitudinal and transverse muscles (*). a, anterior; cm, chaetae protractor muscle; cpm, circular palp musculature; ctm, circular transverse musculature; d, dorsal; dlm, dorsal longitudinal muscles; hm, heart musculature; llm, lateral longitudinal muscles; lm, longitudinal muscles; om, oviduct muscle; p, posterior; rtm, apical ring of transverse muscles; v, ventral; vg, ventral gap of the trunk musculature.

**Figure 6:**
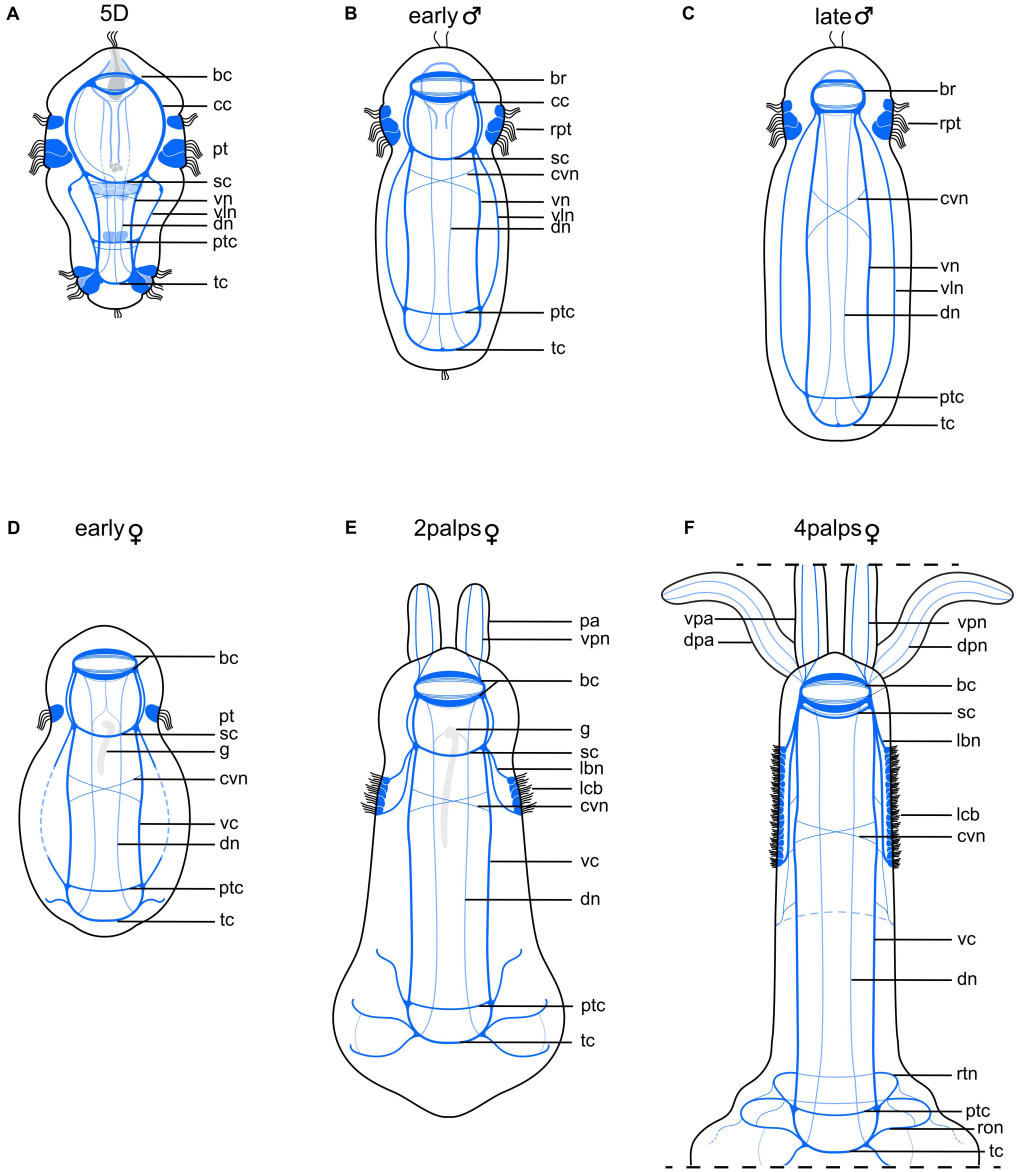
Schematic drawing comparing nervous system of competent larva with those of adult male and female *Osedax japonicus*. **(A)** Nervous system of 5-day old (5D) competent larva. **(B)** Early male nervous system elongates along with body axis elongation. **(C)** Late male nervous system. **(D)** Early female nervous system, still retaining larval structure and “gut” structure (*g*) **(E)** 2-palps female nervous system. Prototroch is fully lost and replaced by innervated lateral ciliary band. **(F)** Nervous system of adult female (4 palps), highlighting increased complexity at both anterior and posterior regions as palps and roots develop, and noting disappearance of gut. Color codes: *dark blue* ventral nervous system structures, *light blue* dorsal nervous system structures. bc, brain commissure; br, brain; cc, circumesophageal connectives; cvn, crossing neurite of the ventral cord; dn, dorsal nerves; dpa, dorsal palp; dpn, dorsal palp nerve; g, ciliated “gut” structure; lbn, longitudinal ciliary band nerve; lcb, longitudinal ciliary band; pa, palp; pt, prototroch; ptc, posterior trunk commissures; ron, ring ovisac net; rpt, reduced prototroch; rtn, ring at the base of the trunk; sc, subesophageal commissure; tc, terminal commissure; vc, ventral cord; vln, ventro-lateral nerves; vn, ventral nerves; vpa, ventral palp; vpn, ventral palp nerve.

Serotonin-like-immunoreactivity and FMRFamide-like immunoreactivity (LIR) are not observed in neurons until 36 h post fertilization, progressively activated throughout the brain and cords during development (Figure 4). Accordingly, the neurogenesis described here relies mainly on observed acetylated α-tubulin-LIR. Additionally, *O. japonicus* displays a distinctive dorsal sensory organ connected to the brain and prototroch, while notably lacking a discernible trochophore ring nerve (Figures 3M,P). During female metamorphosis all of the larval ciliation is lost and additional nerves and muscles develop, whereas males retain most of the larval structures and do not develop any additional nerves or muscles (Figures 5, 6).

### 3.2 Postembryonic staging system for *Osedax* development

#### 3.2.1 Embryo

The early stage development was not studied in detail. Fertilized elliptical yolky eggs spawned by the females were sampled from the mucus holster of the female and isolated for studies of post embryonic development at 10°C in filtered seawater. Within a few hours, the oocyte gained a spherical shape (about 70 μm wide) and underwent first cleavage to the 2-cell stage, from which time the development was recorded as hours post cleavage (hpc). Similar to other studied species of *Osedax* (Rouse et al., 2009), *O. japonicus* exhibits unequal spiral cleavage with the 4-cell stage showing a large “D” quadrant, which during the following cleavage stages results in a large yolky cell constituting the posterior half of the embryo and the early larva (Figure 1, “early embryo”).

#### 3.2.2 1D stage, protrochophore (18+ hpc)

From 18 hpc, the first spherical, slowly and irregular rotating, pre-larva can be recognized (Figure 1, 1D stage). The first 2 rows of sparsely ciliated cells in the equatorial ciliary belt (prototroch) are visible (Figures 2A–C).

A large, and deeply embedded (long necked), flask-shaped cell seems to constitute the center of the apical organ. It shows strong α-tubulin-LIR (Figure 2C), which together with its large posterior body and multiple short cilia may indicate a neuro-secretory function. The first two sparsely ciliated telotroch cells appear. No neural or muscular cells are visible from anti-tubulin or F-actin staining.

#### 3.2.3 2D stage, early trochophore (36+ hpc)

From 36 hpc, the first rotational swimming, non-feeding (lecithotrophic), early trochophore larvae are formed. Their shape is almost spherical, only slightly elongated, with an apical ciliary tuft, a dense prototroch, and a partly developed telotroch (Figure 1, “2D”). The prototroch comprises three rows of closely aligned multiciliated cells, which beat in metachronal waves, generating a forward motion of the larvae in a right-handed helix while rotating around its anterior–posterior axis. The prototroch is still open at the dorsal side (Figure 2E), reflecting the lack of 2d contribution to the prototroch as found in several annelids and mollusks (Nielsen, 2004; McDougall et al., 2006). No eyes are observed, and the larvae show no signs of phototaxis.

The telotroch now comprises about four large and elongated, multiciliated cells, which has thin anterior projections and shows distinct α-tubulin-LIR (Figures 2D,F). The apical organ now comprises a minimum of two additional flask-shaped cells with α-tubulin-LIR but no serotonin- or FMRFamide-LIR (Figure 4). The central nervous system starts developing and is detectable by α-tubulin-LIR. The brain comprises two distinct commissures positioned ventral to the central large flask-shaped cell of the apical organ (Figure 4). Neurons of the brain extend ventrolaterally and posteriorly to form the circumesophageal connectives and into the two ventral cords. The posterior subesophageal commissure is positioned relatively posterior due to the almost spherical shape of the larva, and the two ventral cords are adjoined by anterior projections of two of the telotroch cells (Figure 2D). Two other telotroch cells project antero-laterally, later supporting the ventrolateral nerves (Figure 2F). In the 2D stage, two nerves project posteriorly from the roots of the brain commissures, which in the 3D stage will innervate the dorsal organ. Weak serotonin-LIR is found in the brain commissures and the anterior part of the circumesophageal connectives as well as in two somata of the subesophageal ganglia (Figure 4). FMRFamide-LIR is only detected in two neurons connected to the dorsal organ (Figure 4). However, a strong FRMFamide-LIR is seen in at least five large-bodied epidermal cells located on the ventral apical side, anterior to the prototroch (only shown in 5D, Figures 3F–H). Such large cells with immunoreactive “refringent globules” have also been reported in other larvae such as *Owenia* (Wilson, 1932; Carrillo-Baltodano et al., 2021) and their large-sized cell bodies and strong signal suggest a glandular or potentially neurosecretory function (Wilson, 1932).

No distinct F-actin muscle signal could be detected at this stage.

#### 3.2.4 3D stage, trochophore (60+ hpc)

From 60 hpc, the rotational swimming larvae start elongating and increasing their size (Figure 1). The prototroch is fully developed and continuous across the dorsal midline (Figure 2H). The anteriormost row of prototroch cells have shorter cilia and are separated by one row of nonciliated brown pigmented cells from the two following rows of densely ciliated prototroch cells (Figure 2I). The telotroch cells are fully developed and densely ciliated; however, the ring is not yet closed and thereby has a distinct ventral nonciliated gap. A pair of ventro-lateral protonephridia starts developing posterior to the prototroch (Figure 2G). Yolk cells are concentrated in two medio-centrally located longitudinal bands (Figure 1, “3D”).

Neurons showing α-tubulin-LIR originate at the roots of the brain commissures and encircle the apical organ dorsally. Additional neurons are added to the commissures and the four ventral cords of the central nervous system. The ventrolateral cords are now connected to the brain via the lateral ganglia forming at the level of the subesophageal commissures (Figures 2G,H). Two dorsally positioned telotroch cells project anteriorly, supporting the formation of the two longitudinal dorsal nerves (Figure 2I). Numerous, mono- and multiciliated sensory cells surround the apical organ, almost creating an “apical plate” (Figure 2I). It is not possible to discern which of these cells may functionally belong to the apical organ. However, most of them project axons directly to the brain and none express serotonin- or FMRFamide-LIR (Figure 4). A few neurons seemingly extend laterally from the brain and innervate the prototroch. No prototroch ring nerve could be distinguished.

A likely sensory, dorsal organ develops in a medio-dorsal position, posterior to the prototroch on the hyposphere. This structure is initially visible by α-tubulin-LIR in the cilia. At stage 4-5D, the dorsal organ is comprised of a central group of about eight ciliated cells laterally lined by two collar cells, each with one extremely long (up to 35 μm) stiff, nonmotile cilium (Figures 3M,P). Two distinct dorsal nerves connect the organ with the brain (Figures 3M,P). The first distinct serotonin-LIR is detected in the brain commissures, circumesophageal connectives, subesophageal commissure and in the ventral and ventrolateral nerves. FMRFamide-LIR is seen in the anterior brain commissure and the circumesophageal connectives (Figure 4).

In the 3D stage, the first longitudinal muscle fibers become detectable with F-actin (Figure 5A). At this juncture, the most prominent muscle fibers extend along the dorsal side of the larvae. There are two pairs of dorsal longitudinal muscle fibers, which extend in two directions: from the posterior to the anterior, and from the anterior to the posterior. Additionally, at the anterior end, there is another pair of shorter, dorso-lateral longitudinal muscle fibers. These shorter fibers converge with the longer dorsal fibers and extend posteriorly, though they do not yet extend beyond the prototroch. Two very short lateral longitudinal muscle fibers can sometimes be discerned beneath the prototroch, contributing to the developing musculature (Figure 5A).

#### 3.2.5 4D stage, early metatrochophore (84+ hpc)

From 84+ hpc, the trochophore larva starts developing into a metatrochophore with an oval shape, tapering toward the posterior of the body (Figure 1, “4D”). The two sets of eight hooked chaetae start developing, first showing the approximately 6 teeth of the short hook, later protruding, and the internal shaft thereafter elongating. The 16 larval chaetae are retained into adulthood, and no additional chaetae form. The chaetae are not used for swimming but for crawling and attachment of the metamorphosing larvae. The metatrochophore larva retains occasional helical swimming through metachronal beating of the prototroch, but much of the time the larva undergoes directional fast swimming through synchronous active beating strokes of the prototroch cilia, making the larva move forward in tiny “jumps.” When the larva is physically disturbed, the beating rate of the prototroch is increased, causing fast forward (escape?) movement. The ciliated cells and/or muscle groups are likely controlled separately, since the larva can make fast directional turns when moving up and down sensing the substrate. The swimming movements are likely aided by the telotroch, the three paired multiciliated cells that appear ventrolaterally and later form the medio-anterior transverse ciliary band (paratroch 1), and by the pair of multiciliated cells that appear more posteriorly and medioventrally to later form a medio-posterior transverse ciliary band (paratroch 2). A centro-terminal ciliary tuft (of presumed sensory function) appears within the now fully closed telotroch (Figure 2L), the paired protonephridia are fully developed (Figure 2J), and yolk is further concentrated postero-centrally (Figure 1, “4D”).

In approximately half of the larvae, α-tubulin-LIR revealed a short, anteriorly looped, ciliated tubular structure, which may represent a residual “gut,” present only in female larvae and juveniles (see section 4.1). It has a tiny ventral opening to the external environment, positioned posterior to the prototroch and anterior to the subesophageal commissure (Figure 3H). Additional neurons are added to the commissures and cords of the central nervous system. A ventral terminal commissure of the ventral cords is formed that connects with neurites of the sensory ciliated cells of the centro-terminal tuft. Additional monociliated sensory cells appear anterior and posterior to the prototroch as part of the peripheral nervous system. The axons from these cells either join the ganglia of the cords or extend via the circumesophageal connectives to the brain (Figure 2L). Both serotonin-LIR and FMRFamide-LIR are detected in the ventral nerves and commissures, with FMRFamide-LIR also present in the nerves of the dorsal organ (Figure 4). About 10 somata in the brain and the apical organ exhibit serotonin-LIR, as do six somata of the subesophageal and two of the terminal ganglia; the latter most potentially extend their neurites into the ventrolateral nerves. FMRFamide-LIR appears in two to four somata of the brain (Figure 4).

In 4D larvae, the number of longitudinal muscles along the body increases, reaching a maximum of 16 in some specimens, 8 on each side (Figure 5B). Each of these muscles consists of one to three individual muscle fibers. Notably, the distribution of these muscles along the body is not uniform, with relatively small gaps between them. Additionally, thin transverse circular muscles begin to emerge along the body, forming a delicate muscular mesh.

#### 3.2.6 5D stage, metatrochophore (competent larva; 108+ hpc)

By 108 hpc, the first metatrochophore larvae are fully developed and competent to metamorphose. The 5D larva is slightly pear-shaped with an anterior constriction internal to the prototroch cells and a posterior constriction internal to the telotroch cells (Figure 1, “5D”). This stage retains the dual motility patterns observed in the 4D stage, predominantly exhibiting directional swimming and frequent exploration of the substrate. Bilateral, segmental structures, including chaetae, ciliary bands, commissural somata, and chaetal muscles, are now fully developed. The multiciliated cells of paratroch 1 have fused medially (Figure 3B), and the two rings of each four pairs of chaetae are fully formed. The ciliated “gut” undergoes further development, though it never extends significantly posterior to the level of the subesophageal commissure and never obtains a posterior opening (Figures 3H,J).

Two monociliated apical cells, each with approximately 10 μm long stiff cilia, exhibit dense α-tubulin-LIR. These cells extend contralaterally and posteriorly, seemingly connecting with the brain (Figures 3B,K,L), and may resemble those observed in a similar position in *Capitella* larvae (Meyer et al., 2015). In the competent *O. japonicus* larva, the central nervous system is fully developed, comprising a brain and seven longitudinal nerves: two dorsal nerves, two ventral nerves, two ventro-lateral nerves and a newly formed unpaired medioventral nerve (Figures 3A–D, 4). The ventral nerves are connected by (i) additional neurites of the brain commissures, (ii) additional neurites of the anterior subesophageal commissure, (iii) a posterior trunk commissure (internal to paratroch 2), and (iv) additional neurites of the terminal commissure (Figures 3A,D, 4). Serotonin-LIR is present in additional transverse neurites posterior to the subesophageal commissure, in the two dorsal longitudinal nerves, and the dorsal organ nerves (Figures 3I,J). The number and position of serotonin-LIR somata are similar to those of the 4D stage, with the exception of an additional pair at the roots of the brain commissures and two of the eight somata of the dorsal organ now showing serotonin-LIR (Figures 3I–L). Newly detected neural elements relative to the 4D stage include FMRFamide-LIR in the unpaired medioventral nerve, in two somata of the subesophageal ganglia, and in 16 somata of the potential ganglia of the posterior trunk commissure (Figures 3E–H).

In 5D larvae, the musculature reaches full development, characterized by 16 longitudinal muscle bundles that stretch along the body and slightly taper toward the anterior end (Figures 5B,C). Alongside these longitudinal muscles, a thin mesh of outer circular muscle fibers continues to extend across the body, forming unbroken rings approximately 1 μm apart (Figure 5F). Toward the posterior end, two more distinct rings of these circular muscles become visible. These rings emerge concurrently with the chaetal musculature and demarcate the position of the two chaetae-bearing segments. Additionally, a fully developed chaetal musculature, comprising eight sets, is present. The chaetal musculature remains consistent between the competent larvae and all later stages, except for adult females. Though in males, the spacing between individual muscles increases, making the musculature easier to discern. The chaetal musculature is detailed below.

#### 3.2.7 Early male stage (9+ hpi)

The larvae undergo male metamorphosis very fast, their bodies slightly elongate and round clusters of spermatids become visible with LM from 9 h post incubation (hpi; Figure 1, “early male”). In the presence of females, the males tend to gather around the posterior trunk of the adult female, using their musculature and hooked chaetae to wiggle toward and attach to the female. The early stage males maintain the full prototroch but rapidly lose the telotroch, the apical, terminal, and dorsal organs, along with the paratrochs (Figure 6B). Nerves innervating or extending from these ciliated areas and sensory organs are gradually lost, including the unpaired midventral nerve. Although the posterior trunk commissure is retained, it becomes further distanced from the anterior subesophageal commissure as the trunk elongates (Figure 6B). The sperm development resembles that of other *Osedax* species as described by Worsaae and Rouse (2010). There is no further development or addition of chaetae, muscle bundles, ventral nerves or commissures from the 5D larval stage. The paired protonephridia seem to disappear during metamorphosis.

#### 3.2.8 Late male (96+ hpi)

From 96 hpi, the males contain fully developed sperm, freely dispersed in the body and over time gathering in the dorso-anterior sperm vesicle (Figure 1, “late male”). The prototroch is reduced to a pair of densely ciliated lateral areas, showing a wide dorsal gap and a narrow ventral gap in the previously equatorial ciliation. The body may extend slightly and/or become slenderer as the yolk is used up and the spermatids mature. There are no observed changes in the nervous system during this stage (Figure 6C).

Anteriorly, all of the 16 longitudinal muscle bundles taper and converge, culminating in a connection with a circular muscle constriction. This structure is identified as a ring of transverse muscles (rtm; Figures 5D,G). The rtm seems to function as the attachment site for the longitudinal muscles in both males and females (Figures 5G,H). However, in males, this structure only becomes discernible at later developmental stages. The chaetal musculature represents the most complex element of the muscular system in *O. japonicus* larvae and males, despite the overall simplicity of the larval body. There are eight sets of chaetae in total, with four sets located in each of the posterior two segments. The precise movement of each chaetal set is regulated by its dedicated chaetal musculature. Connected to each chaetal pair are approximately four short muscles that extend from the chaetal sac to the body wall. These muscles are identified as chaetal protractors, which, upon contraction, push the chaetae outward (Figures 5C,G). Additionally, transverse retractor muscles span the chaetae pairs in each hemisegment (Figures 5C,G). These muscles create a connection between the dorsal (notopodial) and ventral (neuropodial) chaetal pairs. When the transverse musculature is contracted, the chaetae are pulled inward, facilitating their retraction. The mature male anatomy and nervous system are highly similar to that of other male *Osedax* described by Worsaae and Rouse (2010).

#### 3.2.9 Early female (9+ hpi)

Female metamorphosis starts with extensive and repeated contractions of the longitudinal musculature, as the females push, wiggle, and attach their posterior bodies into the bony substrate using muscles and chaetae. A thin mucus holster is soon excreted. From nine hpi, early females have distinct short skittle-shaped bodies, retaining only a single row of prototroch cilia. This change involves the loss of two out of three rows of the prototroch, telotroch, paratrochs, and apical, dorsal, and terminal sensory organs (Figure 1, “early female”). The paired protonephridia seem to disappear during metamorphosis, and the internal ciliated “gut” found in some (potential female) larvae extends posteriorly, about halfway of the trunk. The 16 chaetae remain but become progressively less visible as the ovisac and the roots grow. All nerves associated with the lost larval ciliary or sensory structures, as well as the larval latero-ventral nerves, disappear. New peripheral nerves extend laterally from the terminal commissure (Figure 6D).

In early female specimens, the longitudinal muscles show an increase in number, with some individuals exhibiting up to 10 longitudinal muscle bundles extending along the body (Figure 5H). The rest of the musculature is similar to what is observed in competent larvae and males. This includes two transverse bands of circular muscles in the posterior two segments, as well as a clearly discernible ring of transverse muscles (rtm) located anteriorly, serving as the attachment point for the longitudinal muscles. The primary distinction in early females lies in the development of two small palp musculature buds, emerging anteriorly at the dorsal side (Figures 5H,I). Each palp bud encompasses two pairs of longitudinal muscles, which run the length of each palp and appear to connect anterior to the rtm (Figures 5H,I).

#### 3.2.10 2 palp female (36+ hpi)

The females exhibit rapid growth, expanding both in width and length, and after 36 hpi, a beating heart and two palps are discernible dorso-anteriorly with LM (Figure 1, “2-palp female”). The ciliated “gut” extends to about half the trunk length, following the elongation of the trunk (Figure 6E). Two new longitudinal lateral ciliary bands start developing on the trunk. These are innervated by new nerves extending from the brain through the subesophageal ganglia. A pair of nerves extend from the lateral brain into each palp. Additional neurons are added to existing nerves and commissures, and new peripheral nerves extend laterally from the posterior trunk and terminal commissures (Figure 6C). The remaining prototroch disappears.

As the pair of palps in the females become more elongated and prominent, the associated musculature also undergoes further development, with several new additions to the whole muscular system. The longitudinal muscle bands not only increase in number and length but also become denser, encircling the entire trunk (Figure 5K). Concurrently, the number of longitudinal muscle fibers in the palps also increases; however, the total number of four distinct longitudinal muscles within each palp remains constant (Figures 5I,J). This is accompanied by a clearly visible circular musculature that extends along the length of each palp in unbroken rings (Figure 5J). The two rings of posterior transverse circular muscles are still discernible, indicating the position of the chaetae-bearing segments (Figure 5K). Posteriorly, the longitudinal muscles continue their extension. Internally, females begin to develop the muscularized components of the circulatory system. This includes a coiled, U-shaped “heart,” characterized as a tubular structure composed of circular ring muscles. The heart features a tapering anterior end and a broader, open posterior part (Figures 5K,M).

#### 3.2.11 4 palp mature female (2–3 weeks pi)

After 2–3 weeks, the second pair of palps develops dorsal to the first pair, and the three regions of the body are clearly visible: head, trunk, and ovisac/roots (Figure 1, “4-palp female”). Oocytes become visible in the ovisac. The ciliated “gut” is no longer visible, and the subesophageal commissure shifts anteriorly, nearly fusing with the posterior commissure of the brain (Figure 6F). The now fully developed longitudinal lateral ciliary bands have increased in length and width, supported by additional longitudinal nerves that also connect to the ventral cords. The peripheral lateral nerves of the posterior trunk- and terminal commissures now extend to the dorsal side, and seemingly form a continuous ring nerve (Figure 6C). Multiple minor peripheral nerves extend from these and surround the ovisac. The mature female nervous system is highly similar to those of other *Osedax* females described by Worsaae et al. (2016).

In the 4 palp stage, the musculature of the trunk elongates in tandem with the palp musculature extending within the four long palps. Each palp’s musculature is comprised of four longitudinal muscles, each consisting of 3–4 muscle fibers, accompanied by circular muscles that run the entire length of the palp (Figure 5J). This arrangement is consistent with the palp musculature observed in earlier stages, albeit more pronounced. In 4 palp stage females, the longitudinal muscles of the trunk are densely packed and uniformly cover nearly the entire circumference of the trunk, with the exception of a ventral gap (Figure 5N). Internally, a pair of slender longitudinal muscle fibers is situated on each side of the oviduct, extending along its length (Figure 5N). The posterior transverse circular muscles, which previously marked the chaetae-bearing segments, are no longer clearly discernible as the root musculature develops and extends anteriorly. At the transition from the root to the trunk, circular muscle bands become visible as evenly spaced rings, approximately 3 μm apart, with the diameter of these circular muscle fibers being around 0.8 μm (Figures 5N,O). As the root tissue grows, the musculature surrounding the posterior portion of the body undergoes significant changes. Both the circular and longitudinal muscles branch, forming a loosely meshed network that occupies the entire posterior root tissue, resembling a sack (Figure 5O).

## 4 Discussion

### 4.1 *Osedax* exhibits a typical annelid development though lacks a nectochaete stage

It has been suggested that the sex is environmentally determined in *Osedax* (Rouse et al., 2008). Miyamoto et al. (2013) demonstrated that metamorphosis in *O. japonicus* can be initiated by exposing the larvae to various stimuli, including vertebrate bony substrate, symbiotic bacteria, adult females and/or the mucus holster of these females. However, our numerous experiments incubating the competent larvae with different substrates such as fish scales and/or symbiotic bacteria, as well as with females and/or their mucus holster, consistently resulted in a mix of both male and female development. Moreover, about half of the 4-5D larvae developed an internal tubular ciliary structure, which extended in length during female metamorphosis and early stages but was never observed in any males. This finding suggests that sex might be genetically predetermined or determined earlier in larval development than previously suggested (Rouse et al., 2008). Environmental cues appear essential for triggering metamorphosis, but the larvae can survive for at least 3 weeks (at 10°C).

The trochophore larva of *O. japonicus* largely resembles trochophores described for other annelids (Nielsen, 2004). The three closely adjoined ciliary trochs are interpreted as together constituting the prototroch. No adoral ciliation or metatroch exist, reflecting that they are non-feeding, lecithotrophic larvae. However, the internal ciliated structure found in some of the older (potentially female) metatrochophores and in young females might represent a residual gut structure due to its position and configuration. A temporary gut is found in other genera of siboglinids, though in these cases, there is a posterior anus, and a mouth that serves as an entry for uptake of symbionts (e.g., Bright et al., 2013). Further examinations and analyses are necessary to determine the homology and function of this structure in *O. japonicus*. The anterior terminus of the *O. japonicus* “gut” is ventral and posterior to the prototroch, hereby also supporting that the three anterior ciliary bands all are part of the prototroch and that a metatroch is lacking. The two transverse ventral ciliary bandlets of *O. japonicus* show similar late development (in the metatrochophore stage) and position as in *Platynereis* leading us to name these “paratrochs” similar to Fischer et al. (2010), rather than as neuro- or gastrotrochs. In some of the older metatrochophore larvae, smaller groups of cilia are found anterior to the prototroch which might resemble akrotrochs as defined in *Platynereis* (Fischer et al., 2010). However, the *O. japonicus* larvae exhibit an exceptional dorsal sensory organ (see discussion 4.3).

Consistent with previous studies (Rouse et al., 2009; Miyamoto et al., 2013), our findings confirm that the development of *O. japonicus* largely resembles that of pleistoannelids, albeit without a nectochaete stage. We have characterized five postembryonic lecithotrophic larval stages in *O. japonicus* development, which roughly correspond to stages 3–6/7 in *Capitella teleta* (Seaver et al., 2005) and stages 3–7/8 in *Platynereis dumerilii* (Fischer et al., 2010). The larval development of *O. japonicus* terminates sooner than in both these species due to the lack of a nectochaetae stage. Moreover, the competent metatrochophore more closely resembles their early-to mid-metatrochophore stages than their late metatrochophore stage. Annelids generally show three chaetigerous segments in the metatrochophore stage and many more segments in the nectochaete and juvenile stages. *Osedax japonicus* only exhibits two rings of chaetae in the 4-5D metatrochophore stages, and no further chaetae develop in any later stages. Similarly, only two paratrochs and one ventral posterior trunk commissure develop in stage 4-5D, indicating that *Osedax* larvae never develop more than two segments. This early cessation in segmental development align with the fact that juvenile and adult male and female *Osedax* likewise develop no further segments (Worsaae et al., 2016).

### 4.2 *Osedax* myogenesis reveals similarities between metatrochophore- and male myoanatomy

Similar to other annelids, myogenesis in *O. japonicus* initiates with the formation of longitudinal muscle fibers, followed by the development of outer circular musculature and subsequently, the chaetal musculature (e.g., Fischer et al., 2010; Kerbl et al., 2016; Rimskaya-Korsakova et al., 2021). Males of *O. japonicus* maintain the muscular organization of the competent larvae, except for an increased muscle spacing and more pronounced anterior ring of transverse muscles (rtm). This rtm structure was also observed in adult males of other *Osedax* species and aligns with the descriptions provided by Worsaae and Rouse (2010). Similar to males, females also exhibit an anteriorly located rtm, serving as a juncture for both longitudinal trunk muscles and palp musculature. Worsaae and Rouse (2010) suggested that the rtm internally delineates the prostomium. Our observations also support this and imply that the rtm observed in both males and females is homologous.

While the male muscular system largely resembles that of the competent larvae, the muscular system in females exhibits more pronounced transformations following metamorphosis. These changes are chiefly marked by the addition of new muscular structures. Key among these are the circular and longitudinal muscles in the palps, which facilitate palp movement, the muscularized internal heart, oviduct musculature, and in later female stages, a muscularized meshwork throughout the root tissue that develops from branching longitudinal and circular muscles.

### 4.3 *Osedax* neurogenesis reveals similarities between metatrochophore- and male neuroanatomy

The apical organ of *O. japonicus* first becomes visible with α-tubulin-LIR in the 1D protrochophore larva. It comprises a central long-necked, large-bodied, multiciliated, and deeply embedded cell, potentially with neurosecretory function, and at least two adjacent sensory flask-shaped neurons. Neither serotonin-LIR or FMRFamide-LIR are detected in these or other potential α-tubulin-like immunoreactive cells of the apical organ in the early larvae. However, in the 5D larvae, a few centro-anterior cells and their sensory cilia show serotonin-LIR. This indicates that other neurotransmitters of the apical organ and central nervous system are active prior to these common neurotransmitters. Thus, *O. japonicus* does not conform to the suggested conserved pattern of at least two serotonin-like immunoreactive cells in the apical organ of the trochophore (Hay-Schmidt, 2000). Although serotonin-LIR and FMRFamide-LIR are found in the apical organ of multiple larvae, including *Platynereis* (Marlow et al., 2014), the homology or homocracy of neurons, including those showing serotonin-LIR, is highly debated (e.g., Nielsen and Martinez, 2003; Moroz and Romanova, 2023). Moreover, the configuration and appearance of neurons showing serotonin-LIR vary considerably among annelids (Bleidorn et al., 2015; Starunov et al., 2017).

In annelids, the early nervous system has often been found to develop in an anterior to posterior direction, with the first serotonin-like immunoreactive neurons projecting posteriorly from the brain and subesophageal ganglion, forming the ventral cords (e.g., Lacalli, 1984; Hay-Schmidt, 1995; Orrhage and Müller, 2005; Brinkmann and Wanninger, 2008; Bleidorn et al., 2015; Meyer et al., 2015; Kerbl et al., 2016). However, in several annelids, the nervous system is also simultaneously build by posterior neurons projecting anteriorly (Bullock and Horridge, 1965; Dorresteijn et al., 1993; Voronezhskaya et al., 2003; Orrhage and Müller, 2005; McDougall et al., 2006; Fischer et al., 2010; Helm et al., 2013; Bleidorn et al., 2015; Kumar et al., 2020). In the 2D larvae of *O. japonicus*, the earliest detectable neurites of the central nervous system are visible with α-tubulin-LIR and seemingly develop in posterior direction. Serotonin-LIR and FMRFamide-LIR are first found in the anterior nerves of the CNS (in the 2-3D stages) and in two somata of the subesophageal ganglion. Interestingly, α-tubulin-LIR in the posterior telotroch cells reveals an anteriorly directed tube-like extension from their apical side, which seems to connect with the central nervous system at the level of the subesophageal commissure. In a somewhat similar situation, internal extension of multiciliated cells connecting to the ventral nerves have been found in the *Dimorphilus gyrociliatus* dwarf male (Windoffer and Westheide, 1988a,b). Therefore, although no anteriorly growing nerves are detected in the earliest larval stages of *O. japonicus*, the telotroch cells extend anteriorly and potentially aid the later growth of the ventral, ventrolateral, and dorsal nerves along them.

The neural architecture of the competent metatrochophore *O. japonicus* larva overall resembles that of other Pleistoannelida (Bleidorn et al., 2015). It features a pentaneural, ventral nervous system with a brain, circumesophageal connectives, subesophageal and terminal ganglia, but only one posterior pair of trunk ganglia. No stomatogastric nerves were detected in *O. japonicus*, which aligns with the fact that the larva is non-feeding, and the adults lack a gut. Moreover, a prototroch ring nerve could not be detected with serotonin-LIR, FMRFamide-LIR, or α-tubulin-LIR. However, α-tubulin-LIR could have been overlooked due to the strong α-tubulin-LIR of the prototroch cells. It does, though, appear like the sensory cells and nerves of the apical organ, brain and dorsal organ rather connect directly to individual prototroch cells, potentially controlling their movement through different circuits than a prototroch ring.

Trochophore larvae often sense their environment and control their behavior primarily by means of their apical organ (Bleidorn et al., 2015). Our identification of a dorsal organ in *O. japonicus* larvae is unusual and has not been detected in previous studies of other *Osedax* spp. larvae (Rouse et al., 2009), nor is it typical of other annelid larvae (Bleidorn et al., 2015). However, since this structure is not easily visible by LM and it was not detected in a previous LM study of *O. japonicus* (Miyamoto et al., 2013), it may possibly exist in other species of *Osedax*. Temereva and Rimskaya-Korsakova (2023) described a sensory larval structure in the related *Siboglinum fiordicum* Webb, 1963 (Webb, 1963) as a nuchal organ. However, the sensory organ in *O. japonicus* differs vastly in structure and position. Yet, a “dorsal ciliated spot” was reported for the larvae of *S. fiordicum* by Rimskaya-Korsakova et al. (2021), which has a similar position and appearance as the central ciliation of the dorsal organ in *O. japonicus*. Further detailed studies are warranted to test the homology of these siboglinid structures. Interestingly, a highly similar unpaired, mid-dorsal, posterior sensory organ (pso) has been described for the larva of the distantly related annelid *Phyllodoce maculata* (Linnaeus, 1767) (Linnaeus, 1767) (Nezlin and Voronezhskaya, 2003). Such a distinct dorsal ciliated sensory organ has not been described from any other trochophore larvae. Nezlin and Voronezhskaya (2003) notice the strong resemblance of the pso to the apical organ and suggest it to be chemo- or mechanosensory. Similar to our observations, the pso was shown to connect to the brain, the apical organ, and also to the prototroch nerve (Nezlin and Voronezhskaya, 2003). However, contrary to our findings, this organ seemed to disappear in the metatrochophore stage. The very long, stiff cilia of the two collar cells in the dorsal organ of *O. japonicus* suggest a mechanosensory function. However, the dorsal organ may additionally comprise chemosensory cells. It is intriguing to speculate how this sensory structure may regulate larval behavior and co-function with the apical organ.

The metatrochophore nervous system shows great resemblance to that of the male in the presence of a similar number of ventral, ventro-lateral and dorsal nerves and commissures. During metamorphosis, the apical and dorsal sensory organs and most of the larval ciliary bands are lost, along with their associated nerves. The male trunk region is slightly elongated compared to the larva, and with the disappearance of part of the prototroch and the paratrochs during metamorphosis, the subesophageal commissures appear closer to the brain. In Worsaae and Rouse’s (2010) study of *Osedax* males, two anterior commissures were wrongly named a “prototroch ring” commissure (rco). Our study shows that these actually resemble the larval “subesophageal” commissures and are homologous to the anterior commissures in the females, called “cvc” in Worsaae et al. (2016). The crossing ventral nerves (*cvn*) found previously at the anterior trunk of both males and females (Worsaae and Rouse, 2010; Worsaae et al., 2016) are likewise already present in the larva, positioned posterior to the subesophageal commissure. Similar to the posterior trunk commissure, they shift posteriorly as the trunk elongates during male and female development (Figure 6). In summary, no new nerves or commissures of the central nervous system form in the males, and only nerves associated to larval sensory organs and ciliation are lost. In contrast, females develop several new nerves innervating their newly formed palps, lateral trunk ciliation, and the posterior ovisac and roots.

### 4.4 Synchronous arrest in neuro-, myo-, and chaetogenesis during male metamorphosis substantiates the theory of progenesis

Although the metatrochophores of *O. japonicus* is a swimming stage in the life cycle, these larvae are generally observed to be close to the bottom, frequently touching the substrate as if searching for the right place and time to settle. Metamorphosing males, on the other hand, are entirely benthic, stuck within the female’s mucus, using their musculature and chaetae to maneuver toward the female trunk. The similarity in habitat and lifestyle between these two forms, both moving in a biofilm or mucus on large vertebrate bones, may have facilitated the evolutionary transition and success of male miniaturization through progenesis rather than gradual miniaturization. Westheide (1987) similarly proposed that microscopic annelids living between sand grains originated through progenesis rather than gradual miniaturization, as the newly settled larvae or juveniles of their macroscopic annelid ancestor already inhabited the space-restricted and mechanically stressful interstitial environment. Westheide (1987) further argued that natural selection in this habitat might be so harsh that it precludes “middle-sized” body forms, leaving progenesis as the only viable evolutionary “shortcut” for colonizing such selective habitats. The spatially- and temporally limited environment of bony substrates colonized by *Osedax* also appears highly selective, demanding precision in timing, navigation, and motility of small body forms, as well as in fertilization and dispersal strategies (Rouse et al., 2008; Berman et al., 2023). In all studied *Osedax* species, eggs are fertilized before leaving the female oviduct, with internal fertilization likely being essential for ensuring high fertilization rates and successful dispersal and colonization of offspring. Given that *Osedax* lacks a functional gut and can only grow by becoming sessile and developing a root system, we speculate that “middle-sized” males would not increase the male maturation and fertilization rates significantly or reduce inter-gender competition, until the males reach a sufficiently small size that obviates the need for feeding. Moreover, the only known non-dwarf males in *Osedax* are those of *O. priapus,* which has reverted from an ancestral dwarf state (Rouse et al., 2015). Thus, progenesis seems the most plausible “one-step” pathway to a motile, non-feeding, microscopic body form in *Osedax* male.

With the exception of the male reproductive system, our studies of the postembryonic development of *O. japonicus* document that no new organs, segments or even detailed additions to the larval organ systems are formed in the males post metamorphosis. This includes muscular, neural, and chaetal structures that all show a remarkable arrest in somatic development following metamorphosis. The males elongate slightly compared to the metatrochophore larva, and the larval ciliation and associated neurons are lost except for parts of the prototroch that is maintained into adulthood. This strong anatomical resemblance of dwarf males to metatrochophores add to the documentation (Rouse et al., 2009; Worsaae and Rouse, 2010; Miyamoto et al., 2013) that the dwarf males are truly paedomorphic. Moreover, the synchronous arrest in somatic development supports that male dwarfism is caused by the heterochrony process progenesis rather than other miniaturization processes.

While it was not possible to document whether spermatogonia start forming before or during male metamorphosis, spermatids are detectable only a few hours after the onset of metamorphosis, and prior to the loss of larval ciliation. This suggests that rapid sexual maturation not only precedes but potentially plays a role in the synchronous arrest of somatic development in males. Our developmental studies thus substantiate the idea that male dwarfism in *Osedax* is a direct outcome of progenesis (Westheide, 1987), a process hypothesized to involve an early and synchronous cessation of somatic growth, possibly triggered by accelerated or early sexual maturation. Therefore, the miniaturization of *Osedax* males may stand as the most compelling morpho-developmental example to date, validating the previously theoretical process of progenesis as the pathway to a microscopic body plan. This process is initiated within the first hours of metamorphosis. Our newly presented staging system now facilitates further investigations into the genetic distinctions between metatrochophore larvae and metamorphosing males, aiming to uncover the genetic underpinnings of progenesis.

## Data availability statement

The raw data supporting the conclusions of this article will be made available by the authors, without undue reservation.

## Ethics statement

The manuscript presents research on animals that do not require ethical approval for their study.

## Author contributions

KW: Conceptualization, Data curation, Formal analysis, Funding acquisition, Investigation, Methodology, Project administration. Resources, Software, Supervision, Validation, Visualization, Writing – original draft, Writing – review & editing. AR: Data curation, Formal analysis, Investigation, Visualization, Writing – original draft, Writing – review & editing. ES: Formal analysis, Validation, Writing – original draft, Writing – review & editing. NM: Formal analysis, Funding acquisition, Investigation, Resources, Writing – original draft, Writing – review & editing, Validation. ET: Data curation, Formal analysis, Funding acquisition, Investigation, Visualization, Writing – original draft, Writing – review & editing, Validation.
